# Preoperative Risk Assessment and Shared Decision-Making in Older Patients Eligible for Cardiac Surgery: Protocol for a Non-Randomized Prospective Study

**DOI:** 10.3390/healthcare14131900

**Published:** 2026-06-30

**Authors:** Milou S. H. van Dieën, Fredrike Blokzijl, Michiel Kuijpers, Wolter Paans, Maria Agustina Bayon, Suzanne Festen, Willem Dieperink, Wobbe Bouma, Michiel Rienstra, Massimo A. Mariani

**Affiliations:** 1 Research Group Nursing Diagnostics, School of Nursing, Hanze University of Applied Sciences, Petrus Driessenstraat 3, 9714 CA Groningen, The Netherlands; 2 Department of Critical Care, University Medical Center Groningen, University of Groningen, Hanzeplein 1, 9713 GZ Groningen, The Netherlands; 3 Department of Cardiothoracic Surgery, University Medical Center Groningen, University of Groningen, Hanzeplein 1, 9713 GZ Groningen, The Netherlands; 4 Department of Geriatric Medicine, University Medical Center Groningen, University of Groningen, Hanzeplein 1, 9713 GZ Groningen, The Netherlands; 5 Department of Cardiology, University Medical Center Groningen, University of Groningen, Hanzepein 1, 9713 GZ Groningen, The Netherlands; 6 Department of Cardiothoracic Surgery, University Medical Center Utrecht, Heidelberglaan 100, 3584 CX Utrecht, The Netherlands

**Keywords:** decision-making, shared, cardiac surgical procedures, nursing, clinical trial protocol, frail elderly, quality of life

## Abstract

**Background**: The number of older patients undergoing cardiac surgery is increasing, and a substantial proportion of these patients are frail. Frailty is associated with increased mortality, psychological complications, functional decline, and reduced health-related quality of life, complicating treatment decision-making. Currently, treatment recommendations are formulated by the multidisciplinary Heart Team and are mainly based on disease-related parameters. To better integrate patient preferences, geriatric assessment, and shared decision-making into routine cardiothoracic practice, a nurse-led preoperative outpatient pathway was developed: the Preoperative RIsk assessment and shared decision-Making in patients Eligible for cardiac surgery (PRIME) clinic. This study aims to evaluate whether the implementation of PRIME consultation influences Heart Team treatment recommendations and to assess its cost-effectiveness. **Methods**: This single-center, non-randomized prospective study is conducted in the Netherlands. Patients aged ≥70 years who are eligible for cardiac surgery and have two or more risk factors for adverse postoperative outcomes are included. All patients are initially discussed in the Heart Team, resulting in a treatment recommendation. Patients in the intervention group subsequently visit the PRIME clinic, where a comprehensive geriatric assessment and a shared decision-making consultation are performed. Following this visit, patients are re-evaluated by the Heart Team. The primary outcome is the comparison between the initial and subsequent Heart Team treatment recommendations. Secondary outcomes include health-related quality of life and cost-effectiveness. **Discussion**: This prospective exploratory study evaluates the implementation of a novel, patient-centered preoperative care model integrating geriatric assessment and shared decision-making into routine cardiac surgical practice. By focusing on outcomes meaningful to older patients and their relatives, the study contributes to the development of value-based, individualized surgical care. **Trial registration**: The PRIME study was registered in ClinicalTrials.gov (trial registration number: NCT06616129).

## 1. Introduction

The number of older people requiring cardiac surgical interventions is increasing, and a substantial proportion of these individuals are frail [1,2]. Frailty is a syndrome characterized by reduced physiological reserves and impaired ability to maintain internal stability, leading to increased sensitivity to stressors, elevating the risk of adverse health outcomes [3]. Frail patients undergoing cardiac surgery face a significantly higher risk of mortality, prolonged institutional care, and deterioration in health-related quality of life [4]. In addition to surgery-related risks, hospitalization itself may negatively affect older patients, often leading to reduced mobility, loss of muscle strength, and progression of frailty [5]. Furthermore, the impact of cardiac surgery is not limited to cardiovascular factors; it also extends to psychological and emotional dimensions for both patients and their relatives [6,7]. Not only patients, but also relatives face a potentially life-threatening condition. Together, these factors contribute to the complexity and multifaceted nature of determining the optimal treatment plan. While clinical assessment of comorbidities and frailty is essential, patients’ preferences, expectations, and perceptions of health and quality of life should also be considered in treatment decision-making [8].

There is increasing awareness of the benefits of preoperative screening for cardiac surgical decision-making, especially in frail patients [8]. In addition, shared decision-making has been shown to reduce decisional conflict and anxiety, and may influence treatment choices [9,10]. Studies in non-cardiac surgical patients suggest that increased consideration of frailty tends to lead to less invasive treatment [11,12]. However, in the Netherlands, systematic assessment of frailty and patient preferences has not yet been widely implemented in routine cardiac surgical practice. This is likely due to concerns regarding time and resource requirements, as well as uncertainty about how to integrate shared decision-making into standard care [10]. As a result, treatment decisions made during Heart Team meetings, multidisciplinary discussions involving cardiothoracic surgeons and cardiologists, are primarily based on disease parameters.

Given the increasing number of frail older patients who are vulnerable to loss of life and independence, there is a compelling need to organize personalized care for this particular group. Therefore, a recently established outpatient clinic, the Preoperative RIsk assessment and shared decision-Making in patients Eligible for cardiac surgery clinic (PRIME outpatient clinic), was established. This nurse-led PRIME clinic facilitates structured conversations with patients and their relatives, incorporating a comprehensive geriatric assessment and shared decision-making approach to better align treatment decisions with patients’ preferences, expectations, and overall risk profile.

However, the impact of such an approach on treatment decisions and patient outcomes has not yet been formally evaluated. Therefore, the aim of this study is to determine the influence of the PRIME intervention on treatment recommendations made by the Heart Team. Secondary aims are to assess health-related quality of life in patients eligible for cardiac surgery and to evaluate the cost-effectiveness of the PRIME outpatient clinic.

## 2. Materials and Methods

### 2.1. Study Design

This is a single-center, non-randomized, exploratory, prospective trial with data collected during the cardiac treatment decision-making process and a one-year follow-up. Treatment recommendations provided during the first Heart Team meeting (prior to the patient’s visit to the PRIME clinic) will be compared with the (possibly revised) treatment recommendations from the second Heart Team meeting (after the patient’s visit to the PRIME clinic). In addition, patients’ health-related quality of life (measured by RAND-36 [13]) will be assessed at baseline and one year after their visit to the outpatient clinic. These data will be compared with those of a control group with a comparable risk profile who received standard care and did not attend the PRIME clinic.

To evaluate the cost-effectiveness in terms of quality-adjusted life years (QALYs), the EQ-5D-5L [14] and the Medical Consumption Questionnaire [15] (MCQ) will be administered quarterly over a one-year period. This study will be conducted in accordance with the SPIRIT (Standard Protocol Items: Recommendations for Interventional Trials) 2013 statement [16], at the University Medical Center Groningen (UMCG), the Netherlands. The trial is registered at ClinicalTrials.gov, with registration number NCT06616129.

**Figure 1:**
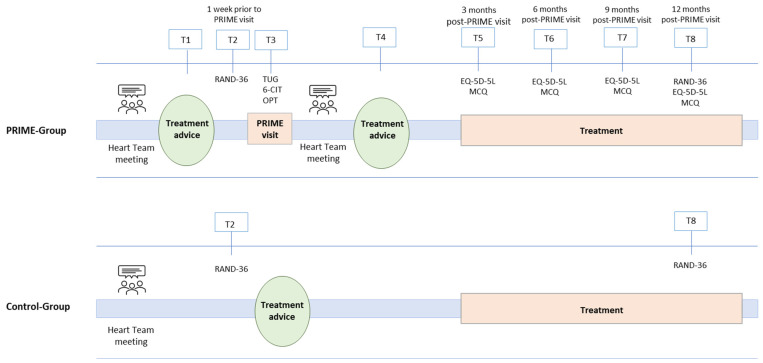
Data collection timeline for the PRIME single-center non-randomized prospective trial.

Figure 1 provides the data collection timeline for the PRIME study design. The Template for Intervention Description and Replication checklist and guide was used to describe the PRIME clinic intervention [17].

### 2.2. Ethics Approval and Informed Consent

The study protocol was approved by the local Medical Ethics Committee (METc number 2022/006) on 10 January 2022 and was deemed not to fall under the scope of the Medical Research Involving Human Subjects Act [18] (WMO).

Written informed consent for participation in the study is obtained from all participants prior to participation. Following enrollment, a unique study identifier is assigned to each participant to ensure confidentiality and data anonymization.

### 2.3. Study Population

One of the major Dutch university heart centers is located at the UMCG, a tertiary referral center [19]. Within the UMCG, referred patients are discussed daily in the Heart Team, which consists of clinical and interventional cardiologists as well as cardiothoracic surgeons. During these meetings, the most appropriate treatment strategy for each patient is determined based on the information provided by the referring cardiologist, including comorbidities and diagnostic test results (e.g., coronary angiography, electrocardiography, and echocardiography).

Patients discussed in the Heart Team will be assessed for eligibility to participate in the PRIME study based on predefined inclusion and exclusion criteria. The inclusion criteria are: age ≥ 70 years, eligibility for cardiac surgery, and having two or more risk factors (see Table 1) associated with adverse outcomes following surgery. These preoperative risk factors include stroke, reduced cognitive function, chronic obstructive pulmonary disease (COPD), obesity, reduced left ventricular ejection fraction, renal failure, reduced mobility, cardiac reoperation, and procedural complexity. The selection of patients with ≥2 risk factors was based on clinical experience and evidence indicating that the accumulation of multiple risk factors is associated with increased postoperative risk in older cardiac surgery patients [20,21,22]. This approach aims to identify a population at higher risk of adverse outcomes, in whom a comprehensive geriatric assessment and shared decision-making are most likely to be clinically relevant.

The control group will consist of elective cardiac patients who received standard care without PRIME consultation. Control patients will be selected from an historical institutional database. To assess whether a tailored treatment plan following PRIME consultation affects health-related quality of life, a control group of approximately 70 control patients will be selected using nearest-neighbor matching. Matching will be based on baseline physical health-related quality of life (RAND-36) and surgical risk as estimated by the EuroSCORE II, to ensure comparable baseline risk profiles and potential for improvement in health-related quality of life [22]. Patients who are unable to read or understand Dutch will be excluded from participation.

### 2.4. Recruitment

The Heart Team refers patients to the PRIME clinic for consultation if they meet the inclusion criteria. The patient will receive an invitation letter approximately 7–10 days prior to their scheduled visit to the outpatient clinic. This invitation includes a letter explaining the purpose of the PRIME clinic visit as well as information about the research being conducted. Patients who wish to participate in the study are requested in the letter to complete the enclosed informed consent form and bring it to their appointment at the PRIME clinic. A screening log is maintained to document the reasons why eligible patients do not participate despite meeting the inclusion criteria.

### 2.5. The PRIME Clinic Intervention

Patients receive treatment advice from the Heart Team and are subsequently referred to the PRIME clinic. During the visit, the patient, along with their family, is seen by a nurse and a nurse practitioner from the Department of Cardiothoracic Surgery. All involved nurses, nurse practitioners, cardiothoracic surgeons, and cardiologists have completed a three-day training ‘Personalized Care and Shared Decision-Making’. The learning objectives of this training included understanding the context, vitality, and frailty of the older patient; identifying the patient’s preferences; estimating the remaining life expectancy and weighing it against the ‘time to benefit’ of a treatment; and systematically devising a tailored treatment plan as a team. Experts from the department of geriatrics and the department of surgical oncology (with experience in similar care transformations) contributed to both the training and the development of the PRIME clinic.

During the PRIME consultation, the nurse and nurse practitioner explain the purpose and approach of the PRIME clinic. Subsequently, the patient and their family member are invited to collaboratively assess treatment choices and clarify the patient’s goals [25]. Alternative treatment options will be discussed, along with their respective pros and cons. During this conversation, the nurse will provide support to the patient, offer health education, and help the patient to elicit informed preferences [25]. Informed preferences revolve around what matters most to the patient and their family.

The Outcome Prioritization Tool (OPT), a conversation aid, is used to elicit the patients’ most important treatment goals. The OPT comprises four universal treatment goals, each rated on a visual analog scale ranging from 0 to 100 [26]. These goals include life extension, maintaining independence, reducing pain, and reducing or eliminating other symptoms. After explanation of these goals, patients are asked to rate each outcome following a structured process: (1) introducing the tool and its rationale; (2) clarification of health-related consequences; (3) explanation of trade-offs, emphasizing the need to prioritize outcomes. If a patient prioritizes one outcome (e.g., prolonging life), this may require compromising on another outcome (e.g., maintaining independence); (4) encouraging the patient to determine the order of importance for health outcomes. In this process, patients are encouraged to articulate their priorities and the reasons behind their choices; and (5) verifying whether the outcomes accurately reflect the patient’s preferences [26].

### 2.6. Preoperative Risk Assessment

During the PRIME consultation, in addition to shared decision-making conversations, a geriatric assessment is conducted. First, all patients referred to the PRIME clinic receive a digital questionnaire. The questionnaire includes items on medical history, comorbidities, medication use, health-related quality of life assessed using the RAND-36 [13,27], and functional status evaluated using the KATZ-ADL questionnaire [28]. A score is calculated for each domain reflecting patients’ perceived health-related quality of life [13].

The nurse and nurse practitioner use the information gathered from the digital or paper questionnaire (depending on the patient’s preference) to prepare for the PRIME consultation.

During the geriatric assessment, patient information is collected across somatic, psychosocial, and functional domains. For the psychological domain, the 6-item Cognitive Impairment Test is used to measure cognitive function [23]. This brief questionnaire consists of six items and is not influenced by the participants’ level of education. Conducting the test takes approximately 3–4 min. The Dutch version of the questionnaire has been validated against the Mini-Mental State Examination [29]. The test includes three items assessing temporal orientation (year, month, time), two tests of attention (counting backwards from 20 to 1 and reciting the months of the year in reverse), and a short-term memory task (recall of a 5-item address). The test uses an inverse scoring system, with weighted items yielding a total score ranging from 0 to 28. Scores of 0–7 are considered normal, while scores of ≥8 indicate cognitive impairment.

Mobility is assessed using the Timed Up and Go test [24,30]. To perform this test, a chair, a stopwatch, and a marked line on the floor are needed. The Timed Up and Go test requires patients to sit on a chair with both hands resting on their thighs if possible. When the nurse gives the starting signal, the patient stands up. The patient then walks as quickly as possible (without running) to the turning point 3 m away from the chair. Beyond the marked line, the patient can choose to turn left or right and walk back to the chair. The stopwatch will be stopped when the initial position is reached. Using a cutoff value of 16 s, the Timed Up and Go test demonstrates high specificity for frailty, with 98 percent of non-frail individuals completing the test in less than 16 s [31].

### 2.7. Multidisciplinary Heart Team Meeting

The nurse practitioner and nurse will discuss the findings of the geriatric assessment, including the patients’ expectations and preferences, during the subsequent multidisciplinary Heart Team meeting. Treatment options are formulated, incorporating the information regarding the patient’s vitality, level of frailty, and aligned with the patient’s goals and priorities. The nurse practitioner informs the patient of the (potentially revised) treatment recommendation from the Heart Team by telephone on the same day.

### 2.8. Outcomes and Measures

Demographic variables include age at the time of the PRIME clinic visit, or at the time of referral to the Heart Team in the control group, as well as sex. Social status at inclusion will be categorized as ‘married or living with a partner’ or ‘unmarried, divorced, or widowed. Education level will be classified according to the Dutch system described by Verhage and the Dutch Central Bureau of Statistics [32], categorizing education into low, medium, and high levels. Relevant comorbidities will be extracted from electronic medical records.

The primary outcome is the difference between the treatment recommendation provided by the multidisciplinary Heart Team prior to the patient’s visit to the PRIME clinic and the recommendation after the PRIME consultation. A revised treatment recommendation is defined as a reconsideration of the initially proposed open-heart surgery discussed during the first Heart Team meeting. In such cases, a less invasive or conservative treatment may be recommended instead of open-heart surgery.

Health-related quality of life will be assessed using the RAND-36 questionnaire [13] in both the intervention and control groups. Measurements will be performed at baseline (prior to the PRIME clinic visit, or 1–4 weeks before surgery in the control group) and at one-year follow-up. The RAND-36 is a validated self-administered questionnaire equivalent to the Short Form-36 (SF-36) for assessing health-related quality of life. It consists of 36 items that evaluate eight health dimensions: physical functioning; role limitations due to physical health; role limitations due to emotional problems; social functioning; emotional well-being; energy levels and fatigue; pain; and general health perceptions [13]. Scores range from 0 to 100, with higher scores indicating better health. Two composite scores can be derived from these domains: a Physical Component Score and a Mental Component Score.

For the economic evaluation, cost-effectiveness will be assessed in terms of quality-adjusted life years (QALYs). The EQ-5D-5L [14] will be used to estimate QALYs, and the Medical Consumption Questionnaire (MCQ) [15] will be used to assess healthcare utilization and associated costs, including hospitalizations, medical procedures, consultations with healthcare providers, medical equipment, and other related expenditures [15]. The EQ-5D-5L and MCQ will be administered only in the intervention group at 3, 6, 9, and 12 months (T5–T8) following the PRIME clinic visit. These measures are not collected in the control group. Healthcare utilization costs will be calculated using standard prices from the Dutch costs’ guidelines [33], and utility values will be calculated based on the current Dutch tariff [34]. See Table 2 for all outcome measures.

Questionnaires will be distributed via email. If patients experience difficulties completing the questionnaires online, paper versions will be provided by post.

### 2.9. Sample Size

Data from a previous study [35] with a similar design, geriatric assessment, and patient age category demonstrated differences in 27% of the treatment recommendations provided by a multidisciplinary team. The primary outcome is a paired binary variable (treatment recommendation before and after PRIME consultation), and differences will be analyzed using McNemar’s test. Accordingly, the sample size calculation was based on McNemar’s test for paired proportions.

To detect a difference of 20%, considered a conservative estimate, with 90% power and a two-sided significance level of 0.05, a sample size of 49 patients is required. The calculation was based on the expected proportion of discordant pairs, assuming that changes would predominantly occur from surgical to conservative recommendations, with minimal reverse changes. To account for potential drop-out and uncertainty in these assumptions, the target sample size was increased to 70 patients.

### 2.10. Statistical Analysis

Patient characteristics will be presented using descriptive statistics, including means (SD) and percentages, as appropriate. The number of revised treatment recommendations due to integration of a geriatric assessment and the decision-making conversation will be presented in percentages.

The RAND-36 will be used to measure health-related quality of life at two time points (T2 and T8) for all participants. The group with revised treatment recommendations will be compared to the group without revised treatment recommendations. Differences in health-related quality of life scores between these two groups at T2 and one-year follow-up (T8) will be analyzed using an independent samples t-test. Additionally, RAND-36 scores of elective cardiac patients with a comparable risk profile who did not attend the PRIME clinic (control group) will be compared with the scores of the PRIME patients.

Results of the economic evaluation will be reported as an Incremental Cost-Effectiveness Ratio, calculated by dividing the difference in effects by the difference in costs. This economic evaluation will be conducted for both patients with consistent treatment recommendations and those with revised treatment recommendations. Differences in health-related quality of life scores and medical costs, measured using the MCQ and EQ-5D-5L at three-month intervals over one year, will be analyzed using a linear mixed effects model. Data analysis will be conducted using IBM SPSS Statistics software, version 28.0 for Windows (SPSS, Inc., Chicago, IL, USA).

### 2.11. Patient and Public Involvement

The national patient association Harteraad [36], which represents the interests of people with cardiovascular diseases, was consulted to provide their perspective on the proposed outpatient PRIME clinic and related study. The design for both the PRIME clinic and the related study has been read, approved, and endorsed by the college of experts with the experience of Harteraad.

## 3. Discussion

The PRIME study will be the first prospective trial to compare cardiac treatment recommendations provided by a multidisciplinary Heart Team before and after a geriatric assessment combined with a structured shared decision-making conversation. In addition, the study will provide insights into patients’ health-related quality of life before and one year after the PRIME consultation and will compare these outcomes with those of a similar group of patients who did not undergo a PRIME consultation. Furthermore, the cost-effectiveness of the PRIME clinic will be evaluated. While the primary goals of cardiac surgery are to improve survival and enhance quality of life [37], previous studies have shown that not all patients benefit from cardiac surgery in terms of improved quality of life [38,39]. Older patients in particular may be at a higher risk of a decline in quality of life and reduced independence following surgery.

Currently, most patients scheduled for cardiac surgery are only seen by healthcare professionals from the cardiothoracic department shortly before surgery. This may lead to late reconsideration of vitality status or operability, requiring valuable time from healthcare providers and causing stress for both patients and their relatives. A key strength of this study is that the implementation of the PRIME clinic enables earlier identification of patient-specific risks and preferences. By integrating this information into the decision-making process, treatment decisions may become more timely and better aligned with patient values, contributing to a more patient-centered approach.

An additional advantage of the PRIME clinic may be the active role of nurses and nurse practitioners in the decision-making process. Despite the recognition of the significance of their role, cardiothoracic nurses and nurse practitioners are currently not involved in the treatment decision-making process, even though they are adequately trained and well-positioned to engage in shared decision-making with patients [40,41].

This single-center exploratory study aims to determine whether a nurse-led outpatient clinic incorporating a shared decision-making conversation and geriatric assessment influences treatment recommendations made by the Heart Team. A potential limitation of this study is the risk of bias in the evaluation of treatment recommendations before and after the PRIME consultation. Both decisions are made within the same institutional setting, and although both Heart Team meetings do not take place on the same day and usually do not involve the same team members, the second evaluation is not formally blinded to the initial recommendation. This may influence the final treatment decision and could bias the primary outcome towards detecting a change. However, blinding of the initial recommendation is not feasible within the current clinical workflow, as this study evaluates a care innovation embedded in routine clinical practice. Future research, preferably in a larger, randomized, multicenter design, could reduce this potential bias and provide more robust evidence regarding the effectiveness of the PRIME intervention.

Before embarking on such larger-scale studies, it is important to assess whether there is sufficient evidence that the PRIME intervention contributes to more personalized care and improves health-related quality of life in frail patients. If the findings of the current study suggest that the PRIME clinic has a meaningful impact on treatment recommendations, this study may serve as a precursor to a future (cluster) randomized controlled trial.

In conclusion, the PRIME study is designed to provide insight into clinical decision-making in cardiac surgery, where shared decision-making and pre-operative geriatric assessment are part of the decision-making process. The PRIME consultation is expected to result in better-informed patients, who are more actively involved in the decision-making process, leading to more value-driven, patient-centered care.

## Figures and Tables

**Table 1:** Definitions of risk factors.

Risk Factor	Definition
Stroke	Transient ischemic attack/cerebrovascular accident [20].
Reduced cognitive function	Problems with a person’s ability to think, learn, remember, use judgment, and make decisions [20]. A score of ≥8 is considered indicative of cognitive impairment, as measured by the 6-item Cognitive Impairment Test [23].
COPD GOLD III/IV	Chronic obstructive pulmonary disease (COPD) is a condition characterized by airflow limitation [21]. In pulmonary function testing, a post-bronchodilator FEV1/FVC ratio below 0.7 is commonly used to diagnose COPD. GOLD III (severe: 30% ≤ FEV1 < 50% of the predicted value) and GOLD IV (very severe: FEV1 < 30% of the predicted value) are considered as risk factors.
Obesity	Body Mass Index > 30 (calculated kg/m^2^) [22].
Left ventricular ejection fraction < 30%	The left ventricular ejection fraction (LVEF) is the primary indicator of left ventricular systolic function [20]. Severe dysfunction measured by ultrasound (LVEF < 30%) is considered a risk factor.
Renal failure	Creatinine > 150 µmol/L [20].
Reduced mobility	Significant limitations in mobility due to musculoskeletal or neurological issues necessitate the use of walking aids or assistance [22]. Mobility measurement is performed using the Timed Up and Go test [24]. A result > 16 s is considered a risk factor.
Cardiac reoperation	A previous history of one or more significant cardiac surgeries that required opening the pericardium [22].
Weight of the procedure	This quantifies the magnitude or scale of the intervention. The reference point is an isolated Coronary Artery Bypass Grafting (CABG) procedure, and procedures considered more complex than this fall into three categories: An isolated non-CABG major procedure (for example, a single valve procedure, replacement of the ascending aorta, correction of septal defects, etc.). Two major procedures (for example, CABG + Aortic Valve Replacement, or CABG + mitral valve repair, or Aortic Valve Replacement + replacement of the ascending aorta, or CABG + maze procedure, etc.). Three major procedures or more (for example, Aortic Valve Repair + Mitral Valve Replacement + CABG, or Mitral Valve Replacement + CABG + tricuspid annuloplasty, etc.), or aortic root replacement when it involves Aortic Valve Repair or repair + coronary reimplantation + root and ascending replacement) [22].

**Table 2:** Outcome measures.

Outcome	Tool	Time Point (T)
Age	Patient files	T2
Sex	Patient files	T2
Living situation	Patient files	T2
Level of education	Patient files	T2
Health-related quality of life	RAND-36	T2, T8
Treatment recommendation before PRIME visit	Patient files	T1
Treatment recommendation after PRIME visit	Patient files	T4
Independence in Activities of Daily Living	Katz Index	T2
Cognitive Impairment Test	6-item Cognitive Impairment Test	T3
Timed Up and Go test	Timed Up and Go test	T3
Outcome Prioritization Tool	Outcome Prioritization Tool	T3
QALYs	EQ-5D-5L	T5–T8
Healthcare use and related medical costs	MCQ	T5–T8

## Data Availability

No new data were created or analyzed in this study. Data sharing is not applicable to this article.

## Abbreviations

The following abbreviations are used in this manuscript:

PRIME	Preoperative RIsk assessment and shared decision-Making in Patients Eligible for cardiac surgery
SF-36	The 36-Item Short Form Health Survey
MCQ	Medical Consumption Questionnaire
EQ-5D-5L	The 5-Level EuroQol 5-Dimension Health Questionnaire
QALY	Quality-Adjusted Life Year
SPIRIT	Standard Protocol Items: Recommendations for Interventional Trials
UMCG	University Medical Center Groningen
6-CIT	6-Item Cognitive Impairment Test
TUG	The Timed Up and Go Test
COPD	Chronic Obstructive Pulmonary Disease
LVEF	Left Ventricular Ejection Fraction
CABG	Coronary Artery Bypass Grafting
OPT	Outcome Prioritization Tool
KATZ-ADL	Katz Index of Independence in Activities of Daily Living
SD	Standard Deviation
T	Time Point
